# Polycomb repressive complex 1 shapes the nucleosome landscape but not accessibility at target genes

**DOI:** 10.1101/gr.237180.118

**Published:** 2018-10

**Authors:** Hamish W. King, Nadezda A. Fursova, Neil P. Blackledge, Robert J. Klose

**Affiliations:** 1 Department of Biochemistry, University of Oxford, Oxford OX1 3QU, United Kingdom

## Abstract

Polycomb group (PcG) proteins are transcriptional repressors that play important roles in regulating gene expression during animal development. In vitro experiments have shown that PcG protein complexes can compact chromatin to limit the activity of chromatin remodeling enzymes and access of the transcriptional machinery to DNA. In fitting with these ideas, gene promoters associated with PcG proteins have been reported to be less accessible than other gene promoters. However, it remains largely untested in vivo whether PcG proteins define chromatin accessibility or other chromatin features. To address this important question, we examine the chromatin accessibility and nucleosome landscape at PcG protein-bound promoters in mouse embryonic stem cells using the assay for transposase accessible chromatin (ATAC)-seq. Combined with genetic ablation strategies, we unexpectedly discover that although PcG protein-occupied gene promoters exhibit reduced accessibility, this does not rely on PcG proteins. Instead, the Polycomb repressive complex 1 (PRC1) appears to play a unique role in driving elevated nucleosome occupancy and decreased nucleosomal spacing in Polycomb chromatin domains. Our new genome-scale observations argue, in contrast to the prevailing view, that PcG proteins do not significantly affect chromatin accessibility and highlight an underappreciated complexity in the relationship between chromatin accessibility, the nucleosome landscape, and PcG-mediated transcriptional repression.

In eukaryotic cells, DNA is wrapped around histone octamers to form nucleosomes and chromatin (Kornberg and Lorch 1999). Chromatin functions to organize the DNA of large eukaryotic genomes into the relatively small confines of the nucleus. The position of nucleosomes on DNA and the organization of nucleosomes into higher-order chromatin structures also plays major roles in gene regulation (Kouzarides 2007; Li et al. 2007). For example, nucleosomes can occlude sequence-specific transcription factors and the transcriptional machinery from accessing the DNA sequence, thus regulating their activity (Kornberg and Lorch 1999; Li et al. 2007; Jiang and Pugh 2009). This can be overcome through the eviction, repositioning, or destabilization of nucleosomes to create chromatin states that are more accessible to *trans*-acting factors (Henikoff 2008; Jiang and Pugh 2009). Accessible chromatin is therefore a characteristic feature of gene regulatory elements including gene promoters and enhancers (Boyle et al. 2008; Song et al. 2011; Thurman et al. 2012). The formation and maintenance of accessible chromatin states appears to be highly regulated, and accessibility is often related to post-translational modification of histones associated with gene regulatory elements. By extension, it has been proposed that chromatin-modifying systems and their associated activities may help to define accessibility at these important regulatory sites.

In animals, Polycomb group (PcG) proteins play central roles in developmental gene regulation. This diverse group of proteins form large multiprotein complexes that bind gene regulatory elements and modify chromatin to establish what is thought to be a transcriptionally repressive chromatin state (Müller and Verrijzer 2009; Di Croce and Helin 2013). PcG proteins generally exist in one of two multiprotein complexes, known as Polycomb repressive complexes 1 and 2 (PRC1 and PRC2). PRC1 complexes, through their catalytic subunit, RING1 (also known as RING1A) or RNF2 (also known as RING1B), monoubiquitylate histone H2A at lysine 119 (H2AK119ub1), whereas PRC2 methylates histone H3 on lysine 27 (H3K27me3). In vertebrates, PRC1 and PRC2 are targeted by various mechanisms to gene promoters, particularly those associated with nonmethylated CpG islands (CGIs) (Bracken and Helin 2009; Simon and Kingston 2013; Blackledge et al. 2015). The occupancy and activity of PcG complexes at CGI gene promoters is typically associated with low or undetectable transcriptional activity. Removal of PcG complexes can lead to the abnormal transcription of PcG-occupied genes (Boyer et al. 2006; Bracken et al. 2006; Endoh et al. 2008; Leeb et al. 2010; Blackledge et al. 2014). Many of these inappropriately activated genes are associated with embryonic development, and their precocious expression during embryogenesis could possibly explain the embryonic lethal phenotypes observed in PcG mutant mice. However, the mechanisms by which PcG complexes achieve transcriptional repression, and how this relates to their activities on chromatin, remain poorly understood.

PcG complexes are thought to repress transcription through the biochemical compaction of chromatin and the creation of inaccessible chromatin at PcG-occupied promoters. This is based in part on in vitro characterization of reconstituted *Drosophila* and mammalian PRC1 complexes which were capable of compacting nucleosomal arrays and inhibiting the activity of nucleosome remodeling complexes (Francis et al. 2004; Trojer et al. 2007, 2011; Grau et al. 2011). These biochemical studies supported a model whereby PcG complexes, particularly PRC1, compact chromatin to create a transcriptionally repressive chromatin state at PcG target sites. In fitting with these in vitro activities, in vivo PcG-occupied promoters also exhibit reduced sensitivity to nuclease digestion when compared to gene promoters lacking PcG complexes (Bell et al. 2010; Calabrese et al. 2012; Kelly et al. 2012; Beck et al. 2014; Deaton et al. 2016). Furthermore, PcG target sites are also more refractory to transcription factor and polymerase binding (Zink and Paro 1995; McCall and Bender 1996; Fitzgerald and Bender 2001). Together, these studies have suggested that DNA within PcG complex-occupied chromatin is less accessible to *trans*-acting factors, consistent with biochemical activities that act locally to compact nucleosomes. However, despite the correlation between PcG protein occupancy and reduced accessibility, and the widespread view that PcG complexes create inaccessible chromatin, genome-scale analyses of whether PcG complexes directly influence chromatin accessibility in vivo remain limited. We therefore have a poor understanding of how chromatin organization is achieved at PcG target sites and how this relates to their repressed transcriptional state.

## Results

### Polycomb-occupied promoters show reduced accessibility

**Figure 1.**
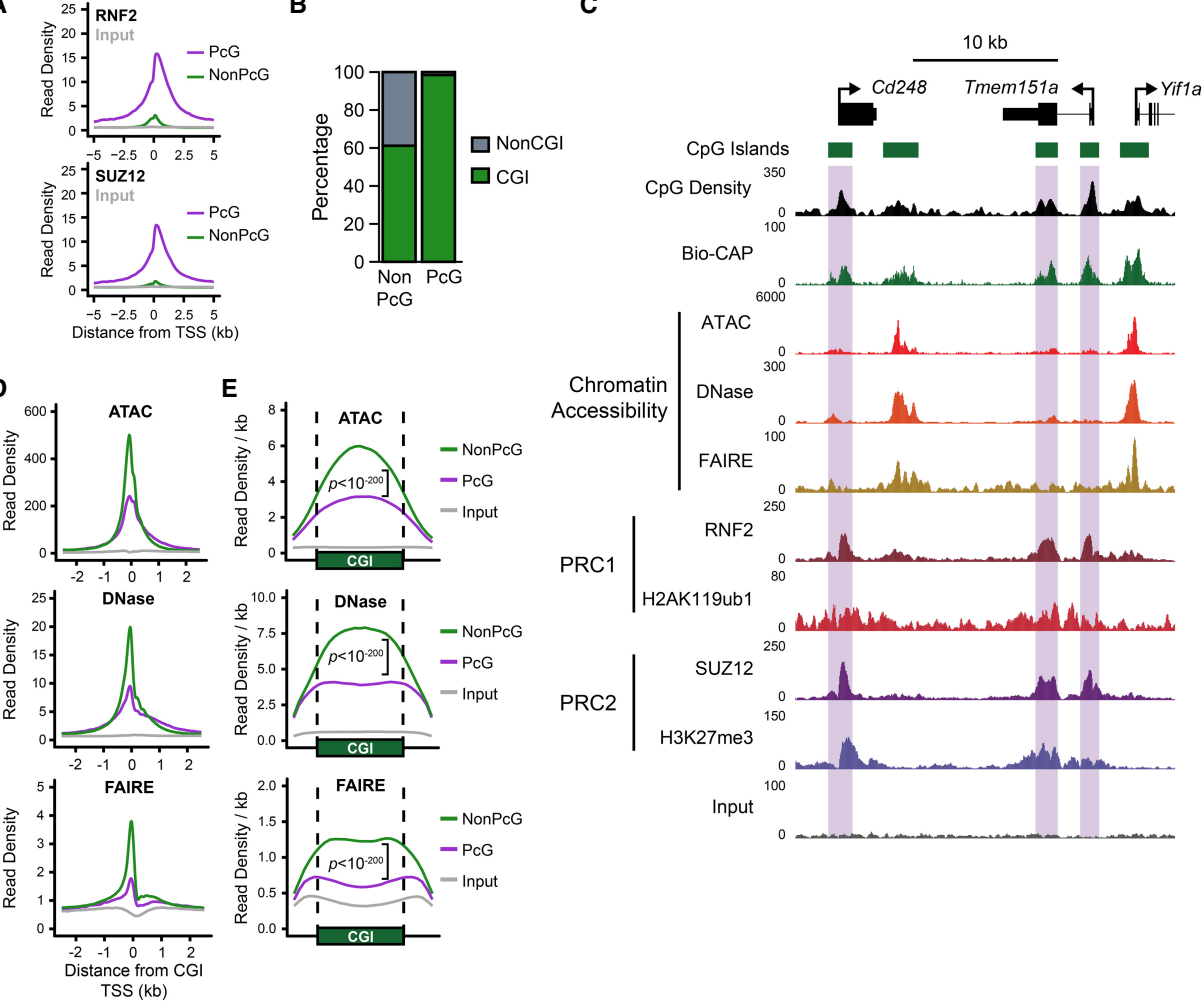
Polycomb-occupied promoters exhibit reduced chromatin accessibility compared to Polycomb-free promoters. (*A*) A metaplot analysis comparing RNF2 (PRC1; *upper*) and SUZ12 (PRC2; *lower*) ChIP-seq signal at Polycomb (PcG)-occupied promoters or PcG-free (non-PcG) promoters in mouse embryonic stem cells (ESCs), centered on transcription start sites (TSSs). (*B*) A comparison of the percentage of PcG and non-PcG TSSs (±500 bp) that overlap with experimentally identified nonmethylated CpG islands (CGIs). (*C*) A genome screenshot of several PcG-occupied promoters (highlighted in purple boxes) profiling three measures of chromatin accessibility: ATAC-seq, DNase-seq, and FAIRE-seq. CpG density and nonmethylated DNA (measured by Bio-CAP), in addition to PRC1 and PRC2 ChIP-seq, are included for reference. (*D*) A metaplot analysis comparing ATAC-seq, DNase-seq, and FAIRE-seq signal at PcG-occupied (*n* = 4020) or PcG-free (*n* = 10,251) CGI promoters, centered on TSSs. Input for ATAC-seq and DNase-seq represents digestion of naked genomic DNA by Tn5 or DNase I, respectively. (*E*) A metaplot analysis at CGI intervals (±20%) for CGI-positive TSSs with (PcG) or without (NonPcG) for ATAC-seq, DNase-seq, and FAIRE-seq signal, normalized to CGI interval size. *P*-values represent comparison of reads per kilobase per million (RPKM) at PcG-bound CGI promoter intervals compared to non-PcG CGI promoters.

Previous studies have demonstrated that PcG proteins and their associated complexes are capable of compacting nucleosomal arrays in vitro (Francis et al. 2004; Trojer et al. 2007, 2011; Grau et al. 2011), whereas in vivo studies have revealed reduced chromatin accessibility at PcG-occupied promoters compared to PcG-free promoters (Bell et al. 2010; Calabrese et al. 2012; Kelly et al. 2012; Beck et al. 2014; Deaton et al. 2016). These nucleosome-based features are distinct from other descriptions of PcG-dependent compaction which occur on the order of ∼100–1000 kb and are more likely to reflect long-range chromatin interactions and higher-order chromatin structures (Eskeland et al. 2010; Schoenfelder et al. 2015; Cruz-Molina et al. 2017; Kundu et al. 2017). To understand whether PcG complexes might indeed regulate the chromatin- and nucleosome-based landscape at gene promoters in living cells, we set out to carefully compare promoters occupied by PcG complexes and those that lack PcG proteins in mouse embryonic stem cells (ESCs) where PcG systems have been extensively studied. To achieve this, we first identified PcG-occupied promoters based on chromatin immunoprecipitation followed by massively parallel sequencing (ChIP-seq) for the PRC1 subunit RNF2 and the PRC2 subunit SUZ12 in mouse ESCs (Fig. 1A). In fitting with previous studies that suggest CpG islands (CGIs) represent an important PcG recruitment site (Mendenhall et al. 2010; Farcas et al. 2012; He et al. 2013; Wu et al. 2013), we observed that 98.4% of PcG-occupied transcription start sites (TSSs) are also marked by the presence of an experimentally identified, nonmethylated CGI (Fig. 1B; Long et al. 2013). Given the substantial differences between CGI and non-CGI chromatin (Blackledge and Klose 2011; Deaton and Bird 2011), we chose to focus our subsequent analysis to CGI-associated gene promoters. Having identified a high-confidence set of PcG-occupied promoters, we then examined whether PcG-occupied gene promoters were associated with chromatin that differed in any way from non-PcG promoters.

We considered three different measures of chromatin accessibility in mouse embryonic stem cells: the assay for transposase accessible chromatin (ATAC-seq), DNase I hypersensitivity (DNase-seq), and formaldehyde-assisted isolation of regulatory elements (FAIRE-seq). ATAC-seq and DNase-seq measure accessibility by interrogating the digestion frequency of chromatin by Tn5 transposase or DNase I, respectively (Cockerill 2011; Buenrostro et al. 2013). Alternatively, FAIRE-seq uses a biochemical approach to purify DNA fragments that are not physically bound by proteins (e.g., nucleosomes or transcription factors), providing a complimentary measure of whether a genomic locus exists in an accessible state (Giresi et al. 2007). Using these measurements, we compared chromatin accessibility at promoters with or without PcG complex occupancy (Fig. 1C–E). Visual examination of several promoters occupied by PcG proteins clearly demonstrated that they had reduced accessibility when compared to neighboring PcG-free promoters (Fig. 1C). Indeed, a genome-wide analysis confirmed that PcG-occupied promoters exhibited significantly lower levels of accessibility than PcG-free promoters (Fig. 1D), confirming and extending previous observations in both *Drosophila* and mammalian cells (Zink and Paro 1995; McCall and Bender 1996; Boivin and Dura 1998; Fitzgerald and Bender 2001; Bell et al. 2010; Calabrese et al. 2012; Beck et al. 2014; Deaton et al. 2016). This difference in accessibility was not limited to the TSS itself, but instead occurred across the entire breadth of the CGI and its associated PcG chromatin domain (Fig. 1E). Furthermore, PcG complex occupancy was associated with reduced chromatin accessibility when expression-matched PcG and non-PcG promoters were compared (Supplemental Fig. S1A). We also examined the relationship between PcG chromatin domains and promoter accessibility in other mouse tissues and cell lines. We found that reduced chromatin accessibility at PcG promoters was widespread (Supplemental Fig. S1B,C). Although PcG complex enrichment is usually associated with gene promoters in mammalian cells, we also examined the subset of distal elements bound by PcG complexes and compared them to distal elements that lacked PcG complexes (Supplemental Fig. S2A,B). In contrast to gene promoters, PcG-bound distal elements showed little difference in their accessibility when compared to distal elements without PcG binding. This suggested that PcG complexes may specifically limit chromatin accessibility at gene promoters.

### Elevated occupancy and closer spacing of nucleosomes at PcG-occupied promoters

**Figure 2.**
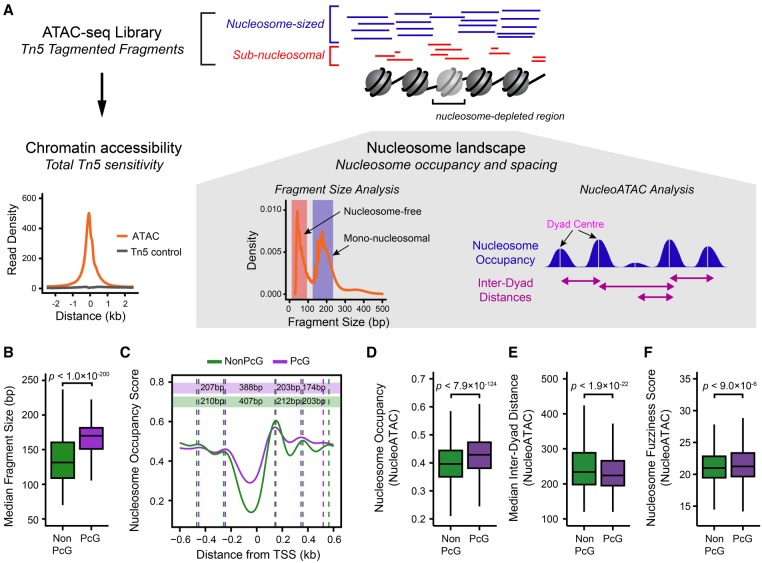
Characterization of the nucleosome landscape at Polycomb-occupied promoters. (*A*) A schematic detailing the approach to analyze nucleosome landscape features from ATAC-seq data. The cleavage of Tn5 hypersensitive DNA (accessible DNA) by Tn5 generates DNA fragments that broadly reflect either mononucleosomal fragments (blue) or nucleosome-free fragments (red). The total count of fragments represents total chromatin accessibility at a given loci, and the fragment size distribution allows the examination of qualitative features of Tn5 sensitivity, such as nucleosome occupancy or positioning using either the median fragment size for a gene promoter or the quantification of nucleosome occupancy signal using the software package NucleoATAC (Schep et al. 2015). After identifying nucleosome positions using NucleoATAC, individual nucleosome dyad centers can then be identified, and the distance between neighboring dyad centers can be calculated. (*B*) A box plot comparing the median ATAC-seq fragment sizes for PcG-occupied (*n* = 4020) or non-PcG (*n* = 10,251) CGI promoters. PcG-occupied promoters tend to have larger fragment sizes consistent with an enrichment for nucleosomal-sized fragments. (*C*) A metaplot for PcG-occupied or PcG-free promoters depicting nucleosome occupancy signal extracted from ATAC-seq data using NucleoATAC, centered on TSSs. The average dyad center for each nucleosome position is marked by dashed lines, and the distance between each nucleosome position is included in the colored rectangles: (purple) PcG; (green) NonPcG. (*D*) A box plot comparing the NucleoATAC-derived nucleosome occupancy score within PcG-occupied or PcG-free promoters. (*E*) A box plot comparing the median inter-dyad distances within PcG-occupied or PcG-free promoters. Distances were calculated between the centers of neighboring dyad positions identified by NucleoATAC. (*F*) A box plot comparing the median fuzziness score for nucleosomes identified by NucleoATAC within PcG-occupied or PcG-free promoters.

PcG promoters have been proposed to exist in a more nucleosome-enriched state compared to non-PcG promoters in mammalian cells (Kelly et al. 2012; West et al. 2014). We were therefore keen to explore in more detail the nucleosome landscape at PcG-occupied gene promoters in our ATAC-seq experiments. To achieve this, we extracted nucleosome occupancy and positioning data using the NucleoATAC approach (Fig. 2A; Schep et al. 2015) and compared PcG-occupied promoters and PcG-free promoters in mouse embryonic stem cells (Fig. 2B–F). Here, we use the term nucleosome occupancy to describe the observed level of mononucleosome signal at defined nucleosome dyad centers, whereas nucleosome spacing refers to the distance between identified nucleosome positions (Fig. 2A; for more details, see Methods). Our analysis revealed elevated nucleosome occupancy at PcG promoters compared to PcG-free promoters, in agreement with previous observations (Fig. 2B–D; Kelly et al. 2012; West et al. 2014). However, PcG-bound TSSs are still depleted of nucleosomes compared to the surrounding genome (Hagstrom et al. 1997; Mohd-Sarip et al. 2005, 2006; Papp and Müller 2006; Mito et al. 2007). One of the proposed functions of PcG complexes is to compact nucleosomal arrays (Francis et al. 2004; Trojer et al. 2007, 2011; Grau et al. 2011). We therefore examined the spacing between nucleosomes at PcG-occupied promoters and observed that nucleosomes at PcG-occupied promoters exhibited shorter inter-dyad distances and their positions were less well-defined when compared to nucleosomes found at PcG-free promoters (Fig. 2C,E,F). Elevated nucleosome occupancy and less well-positioned nucleosomes were clearly evident at PcG-bound promoters across all expression quantiles (Supplemental Fig. S3A), as well as at PcG-bound distal regulatory elements (Supplemental Fig. S2B). Together, these observations indicate that PcG target sites in ESCs exist in a nucleosome-rich state with more closely spaced nucleosomes than PcG-free regions.

### Deletion of PRC1, but not PRC2, results in altered nucleosome occupancy and spacing without changes in chromatin accessibility

**Figure 3.**
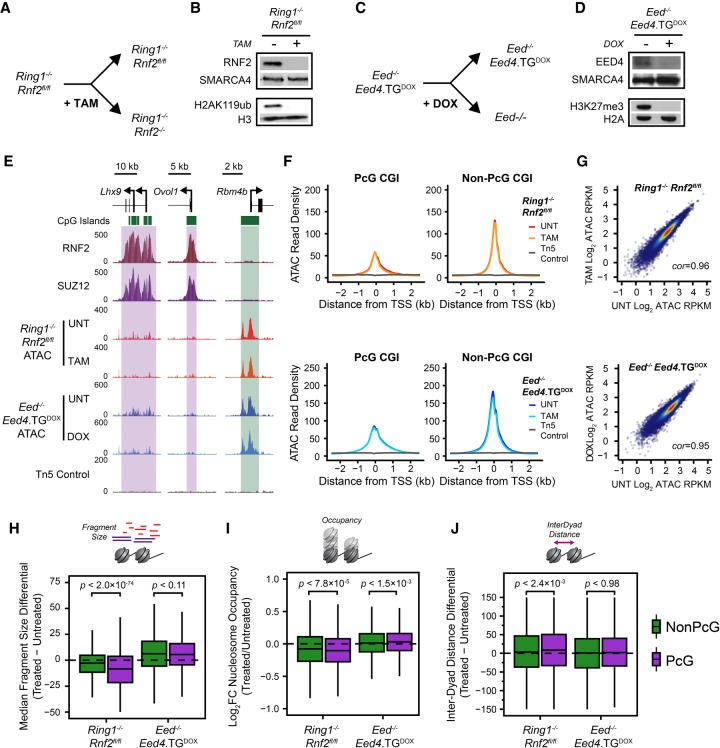
PRC1 contributes toward nucleosome spacing and occupancy but not chromatin accessibility. (*A*) A schematic depicting the treatment of *Ring1*^−/−^;*Rnf2*^*fl/fl*^ ESCs with 4-hydroxytamoxifen (TAM) to generate PRC1-null ESCs. (*B*) A Western blot analysis of untreated and TAM-treated *Ring1*^−/−^;*Rnf2*^*fl/fl*^ ESCs for RNF2 and H2AK119ub1. (*C*) A schematic depicting the treatment of *Eed*^−/−^;*Eed4.TG*^*DOX*^ ESCs with doxycycline (DOX) to generate PRC2-null ESCs. (*D*) A Western blot analysis of untreated and DOX-treated *Eed*^−/−^;*Eed4.TG*^*DOX*^ ESCs for EED and H3K27me3. (*E*) Genome screenshots of chromatin accessibility, as measured by ATAC-seq, at two PcG-occupied promoters (purple boxes) and one PcG-free promoter (green box) before and after conditional deletion of PRC1 or PRC2 from mouse ESCS. (*F*) A metaplot analysis for *Ring1*^−/−^;*Rnf2*^*fl/fl*^ and *Eed*^−/−^;*Eed4.TG*^*DOX*^ ATAC-seq before and after TAM or DOX treatment, respectively, at PcG-occupied (*n* = 4020) or non-PcG CGI promoters (*n* = 10,251), centered on TSSs. (*G*) A scatterplot analysis comparing untreated and treated reads per kilobase per million (RPKM) for *Ring1*^−/−^;*Rnf2*^*fl/fl*^ and *Eed*^−/−^;*Eed4.TG*^*DOX*^ ATAC-seq at all CGI promoters. (*H*) A box plot comparing the change in median Tn5-tagmented fragment sizes for PcG and non-PcG CGI promoters in *Ring1*^−/−^;*Rnf2*^*fl/fl*^ and *Eed*^−/−^;*Eed4.TG*^*DOX*^ ATAC-seq data sets before and after TAM or DOX treatment, respectively. A decrease in median fragment size reflects a shift toward a more nucleosome-free state. (*I*) A box plot quantifying the log_2_ fold change (log_2_FC) in NucleoATAC-derived nucleosome occupancy for PcG and non-PcG CGI promoters in *Ring1*^−/−^;*Rnf2*^*fl/fl*^ and *Eed*^−/−^;*Eed4.TG*^*DOX*^ ATAC-seq data sets before and after TAM or DOX treatment, respectively. (*J*) A box plot comparing the difference in median inter-dyad distances for PcG and non-PcG CGI promoters in *Ring1*^−/−^;*Rnf2*^*fl/fl*^ and *Eed*^−/−^;*Eed4.TG*^*DOX*^ ATAC-seq data sets before and after TAM or DOX treatment, respectively.

Our characterization of the chromatin landscape at PcG-occupied promoters is consistent with previous reports implicating PcG complexes in the compaction of nucleosome arrays to create inaccessible chromatin. However, whether PcG complexes themselves define these features in vivo has yet to be interrogated satisfactorily. We therefore set out to examine the chromatin landscape of PcG-occupied promoters in cells lacking normal PcG complex activity (Fig. 3). To achieve this, we exploited mouse ESC lines to ablate either PRC1 or PRC2. We used *Ring1*^−/−^;*Rnf2*^*fl/fl*^ conditional ESCs (Endoh et al. 2008) in which the addition of tamoxifen leads to *Rnf2* deletion and the creation of PRC1-null cells (Fig. 3A). As expected, treatment of this cell line with tamoxifen was sufficient to remove RNF2 protein and PRC1-deposited H2AK119ub1 (Fig. 3B). Alternatively, the PRC2 core complex was removed using an EED conditional knockout cell line (*Eed*^−/−^;*Eed4.TG*^*DOX*^) which expresses a doxycycline-sensitive *Eed4* transgene (*Eed4*^TG^) in an *Eed*^−/−^ background (Fig. 3C; Ura et al. 2008). In the presence of doxycycline, *Eed4*^TG^ is not expressed, leading to loss of EED expression, destabilization of the core PRC2 complex (Ura et al. 2008; Tavares et al. 2012), and loss of H3K27me3 (Fig. 3D). We performed ATAC-seq in the *Ring1*^−/−^;*Rnf2*^*fl/fl*^ and *Eed*^−/−^;*Eed4.TG*^*DOX*^ ESCs in order to understand whether PRC1 or PRC2 are responsible for the chromatin features associated with PcG occupancy in mouse ESCs. Initially we considered two PcG-occupied genes, *Lhx9* and *Ovol1*, with low chromatin accessibility at their promoters in wild-type ESCs and examined their accessibility in the PRC1- or PRC2-null state (Fig. 3E). There was no apparent change in chromatin accessibility in the absence of either PRC1 or PRC2 at these loci. We then extended this analysis across all PcG-occupied promoters. Again, we did not identify any significant changes in chromatin accessibility following deletion of either PRC1 or PRC2 (Fig. 3F,G; Supplemental Fig. S4), in agreement with a previous study examining chromatin accessibility in the *Ring1*^−/−^;*Rnf2*^*fl/fl*^ ESCs (Hodges et al. 2018). This was unexpected given the previously observed biochemical activities of PcG complexes, therefore revealing that deletion of PRC1 or PRC2 does not influence chromatin accessibility at PcG-occupied gene promoters in ESCs. To determine whether this was also the case in other cell types, we examined chromatin accessibility in a *Ring1*^−/−^;*Rnf2*^*fl/fl*^ conditional mouse embryonic fibroblast cell line by ATAC-seq (Supplemental Fig. S5A–C). In wild-type fibroblasts PcG-occupied promoters exhibited lower levels of chromatin accessibility compared to PcG-free promoters. In agreement with our analysis of PRC1-null ESCs, this disparity between chromatin accessibility of PcG-bound promoters and PcG-free promoters remained after deletion of PRC1 in the *Ring1*^−/−^;*Rnf2*^*fl/fl*^ fibroblasts (Supplemental Fig. S5C), although we did observe modest and nonspecific increases in accessibility more generally. Therefore, we conclude that although PcG-bound promoters are associated with reduced accessibility compared to PcG-free promoters, PcG systems do not directly create this lack of accessibility.

Because PcG-occupied gene promoters also show elevated nucleosome occupancy and closer nucleosome spacing (Fig. 2), we were keen to examine whether these nucleosome features might be altered in the absence of either PRC1 or PRC2 (Fig. 3H–J). These analyses revealed that the deletion of PRC1, but not PRC2, resulted in reductions in the mononucleosome-sized fragments and NucleoATAC-derived nucleosome occupancy scores (Fig. 3H,I; Supplemental Fig. S6A) coupled with subtle increases in inter-nucleosomal spacing (Fig. 3J) at PcG-occupied promoters. We also performed MNase-seq as an alternative measurement of nucleosome positioning and occupancy in the *Ring1*^−/−^;*Rnf2*^*fl/fl*^ ESCs (Supplemental Fig. S6B–D) and examined ATAC-derived nucleosome features in *Ring1*^−/−^;*Rnf2*^*fl/fl*^ mouse embryonic fibroblasts (Supplemental Fig. S5D). These analyses revealed similar reductions in nucleosome occupancy and altered nucleosome spacing in the absence of PRC1, although this was less apparent in mouse embryonic fibroblasts. Importantly, these effects were not observed or considerably less dramatic at non-PcG promoters, indicating that this effect was specific to the promoters occupied by PcG complexes. To our knowledge, these observations demonstrate for the first time in vivo that PRC1 can influence the nucleosome landscape by altering nucleosome occupancy and spacing, albeit modestly, in a way that does not appear to define overall accessibility at the gene promoters. This suggests that these features are not directly coupled at PcG-occupied gene promoters.

### PcG complexes do not function redundantly to shape the chromatin landscape at PcG-occupied promoters

**Figure 4.**
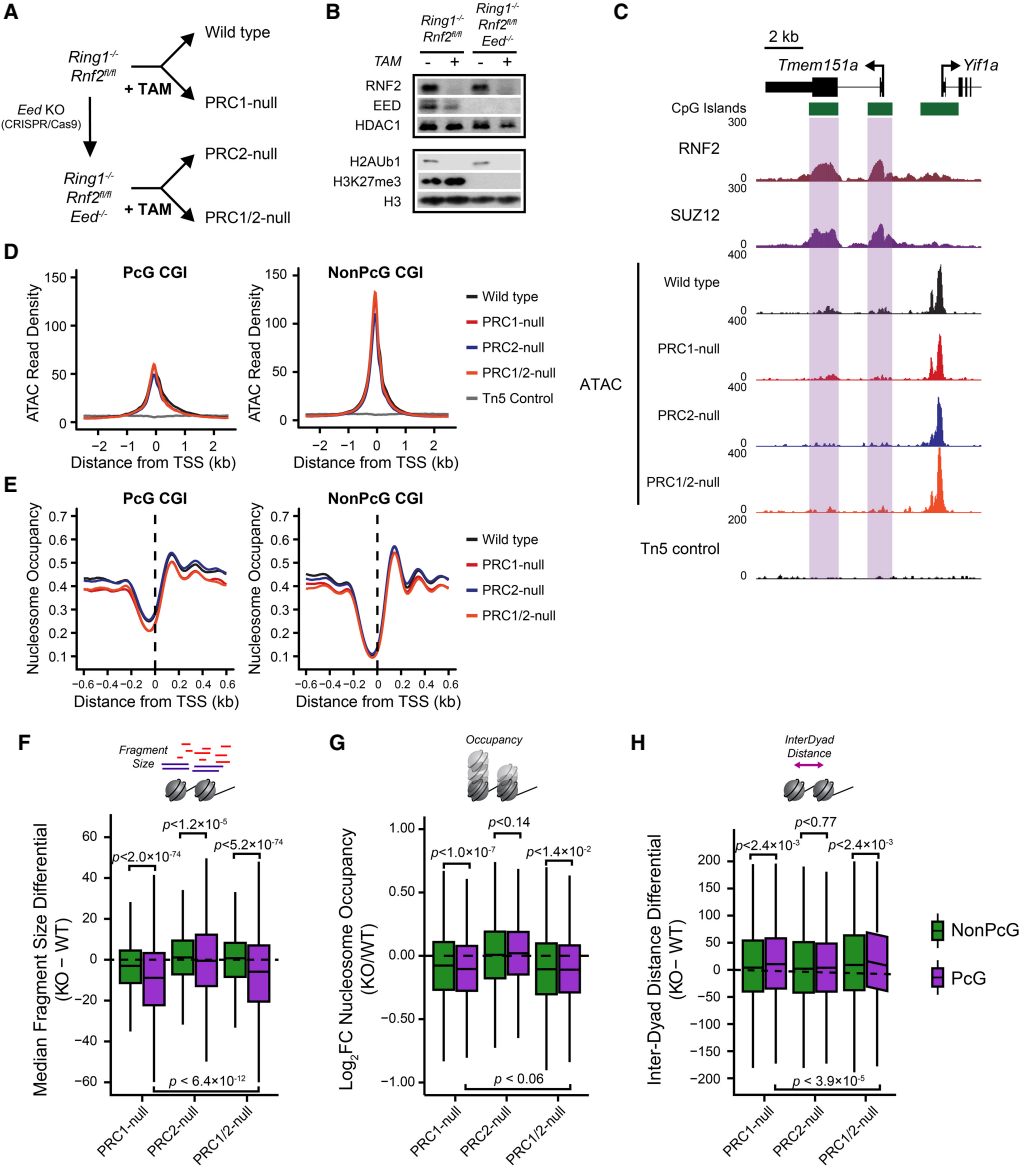
PRC1 and PRC2 do not function redundantly to shape the chromatin landscape at PcG-occupied gene promoters. (*A*) A schematic detailing the strategy to ablate PRC1 and/or PRC2 in mouse ESCs. (*B*) A Western blot analysis for RNF2, EED, H3K27me3, and H2AK119ub1 for *Ring1*^−/−^;*Rnf2*^*fl/fl*^ and *Ring1*^−/−^;*Rnf2*^*fl/fl*^;*Eed*^−/−^ ESCs with or without tamoxifen (TAM) treatment after 72 h. (*C*) A genome screenshot for *Ring1*^−/−^;*Rnf2*^*fl/fl*^ and *Ring1*^−/−^;*Rnf2*^*fl/fl*^;*Eed*^−/−^ ATAC-seq signal before and after TAM at PcG-occupied CGIs (highlighted in purple) and a non-PcG CGI promoter. (*D*) A metaplot analysis for *Ring1*^−/−^;*Rnf2*^*fl/fl*^ and *Ring1*^−/−^;*Rnf2*^*fl/fl*^;*Eed*^−/−^ ATAC-seq before and after TAM treatment at PcG-occupied (*n* = 4020) or non-PcG CGI promoters (*n* = 10,251), centered on TSSs. (*E*) A metaplot analysis for *Ring1*^−/−^;*Rnf2*^*fl/fl*^ and *Ring1*^−/−^;*Rnf2*^*fl/fl*^;*Eed*^−/−^ NucleoATAC-derived nucleosome occupancy score before and after TAM treatment at PcG-occupied or non-PcG CGI promoters, centered on TSSs. (*F*) A box plot comparing the change in median Tn5-tagmented fragment sizes for PcG and non-PcG CGI promoters between *Ring1*^−/−^;*Rnf2*^*fl/fl*^ and *Ring1*^−/−^;*Rnf2*^*fl/fl*^;*Eed*^−/−^ATAC-seq data sets before and after TAM treatment. (*G*) A box plot quantifying the log_2_ fold change (log_2_FC) in NucleoATAC-derived nucleosome occupancy for PcG and non-PcG CGI promoters in *Ring1*^−/−^;*Rnf2*^*fl/fl*^ and *Ring1*^−/−^;*Rnf2*^*fl/fl*^;*Eed*^−/−^ ATAC-seq data sets before and after TAM treatment. (*H*) A box plot comparing the difference in median inter-dyad distances for PcG and non-PcG CGI promoters in *Ring1*^−/−^;*Rnf2*^*fl/fl*^ and *Ring1*^−/−^;*Rnf2*^*fl/fl*^;*Eed*^−/−^ ATAC-seq data sets before and after TAM treatment.

Previous studies have identified some instances of redundancy between the activity and function of PRC1 and PRC2 (Leeb et al. 2010). Furthermore, deletion of PRC1 results in widespread reductions, but not complete loss, of PRC2 at PcG-occupied promoters (Blackledge et al. 2014), and vice versa in PRC2-null cells (Tavares et al. 2012). As such it seemed possible that redundancy between PRC1 and PRC2 could potentially mask any effects on chromatin accessibility or more profound effects on nucleosome features at PcG-occupied promoters. We therefore sought to develop a cell culture system in which we could remove both PRC1 and PRC2. Previous reports have established that mouse ESCs lacking PRC2 are viable and can be maintained in culture (Boyer et al. 2006; Chamberlain et al. 2008; Shen et al. 2008; Leeb et al. 2010), whereas cells lacking PRC1 differentiate and are unable to be maintained as pluripotent cells (Stock et al. 2007; Endoh et al. 2008). Therefore, we constitutively deleted *Eed* (EED) in the *Ring1*^−/−^;*Rnf2*^*fl/fl*^ conditional ESCs using CRISPR/Cas9-mediated gene editing (Fig. 4A) and confirmed loss of EED protein levels by Western blotting (Fig. 4B). Treatment of *Ring1*^−/−^;*Rnf2*^*fl/fl*^;*Eed*^−/−^ ESCs with tamoxifen resulted in the complete loss of PRC1, effectively removing both PcG complexes (Fig. 4B). We then examined the chromatin landscape at PcG-occupied gene promoters by performing ATAC-seq on the *Ring1*^−/−^;*Rnf2*^*fl/fl*^;*Eed*^−/−^ ESCs with or without tamoxifen treatment. Even in the absence of both PRC1 and PRC2, there were no increases in accessibility at either individual PcG-occupied promoters (Fig. 4C) or genome-wide (Fig. 4D), similar to our observation in lines with deletion of PRC1 or PRC2 individually. However, consistent with a role for PRC1, but not PRC2, in modulating nucleosome spacing and occupancy at PcG-occupied promoters, we observed a shift toward nucleosome-free DNA and reduced nucleosome occupancy (Fig. 4E–G), as well as increased inter-dyad spacing (Fig. 4H), at PcG-bound promoters only in the PRC1- and PRC1/2-null cells. The effects in PRC1/2-null cells were similar to those observed in cells lacking only PRC1 (Fig. 4E–H), suggesting little if any contribution of PRC2 to the regulation of nucleosome occupancy and spacing at PcG target sites, although we cannot exclude the possibility that this may reflect an adaptation of the nucleosome landscape in these cells due to constitutive loss of PRC2. Importantly, the fact that neither PRC1 or PRC2 appear to be responsible for limiting chromatin accessibility of PcG-occupied gene promoters in ESCs suggests that other pathways or processes must determine the reduced accessibility at these sites (Discussion).

### Remodeling of the PRC1-dependent nucleosome landscape is linked to RNA polymerase II activity

**Figure 5.**
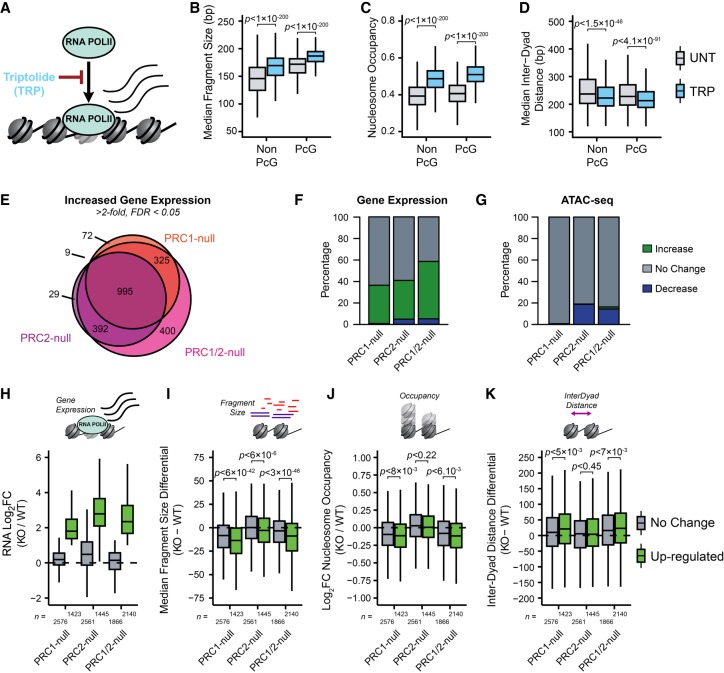
The PRC1-dependent nucleosome landscape is linked to, but not explained by, RNA polymerase II activity. (*A*) A schematic depicting the inhibition of RNA polymerase II (RNA POLII) occupancy using triptolide (TRP). (*B*) A box plot comparing the median Tn5-tagmented fragment sizes for PcG and non-PcG CGI promoters before and after TRP treatment. (*C*) A box plot quantifying the NucleoATAC-derived nucleosome occupancy score for PcG and non-PcG CGI promoters before and after TRP treatment. (*D*) A box plot comparing the median inter-dyad distances for PcG and non-PcG CGI promoters before and after TRP treatment. (*E*) A Venn diagram for PcG-occupied CGI promoters with significant increases in gene expression in *Ring1*^−/−^;*Rnf2*^*fl/fl*^ and *Ring1*^−/−^;*Rnf2*^*fl/fl*^;*Eed*^−/−^ ATAC-seq before and after tamoxifen (TAM), corresponding to PRC1-null, PRC2-null, and PRC1/2-null (FDR < 0.05; fold change > 2). (*F*) A bar plot depicting the number of significant RNA-seq expression changes for PcG-occupied CGI promoters. (*G*) Same as in *F*, only for ATAC-seq. Changes in ATAC-seq were calculated using the CGI promoter interval. (*H*) A box plot comparing the change in RNA-seq log_2_ fold change (log_2_FC) for PcG-occupied CGI promoters with (up-regulated) or without (no change) an increase in gene expression for each cell line and treatment. (*I*) A box plot comparing the change in median Tn5-tagmented fragment sizes for PcG-occupied CGI promoters with (up-regulated) or without (no change) an increase in gene expression for each cell line and treatment. (*J*) A box plot quantifying the log_2_ fold change (log_2_FC) in NucleoATAC-derived nucleosome occupancy for PcG-occupied CGI promoters with (up-regulated) or without (no change) an increase in gene expression for each cell line and treatment. (*K*) A box plot comparing the difference in median inter-dyad distances for PcG-occupied CGI promoters with (up-regulated) or without (no change) an increase in gene expression for each cell line and treatment.

PcG complexes are required to maintain a transcriptionally repressive chromatin environment at developmentally regulated gene promoters. Our characterization of the nucleosome landscape revealed that PRC1 plays a unique role in shaping nucleosome occupancy and spacing at PcG-occupied promoters and that in the absence of PRC1 this PcG-associated nucleosome landscape reverted to an arrangement reminiscent of more transcribed non-PcG-associated promoters (Fig. 4). We therefore hypothesized that this altered nucleosome landscape may manifest not simply from the absence of PRC1 but instead as a result of activation of genes normally occupied by PcG complexes, potentially as a direct consequence of increased RNA polymerase II activity (Kireeva et al. 2002; Gilchrist et al. 2010; Kulaeva et al. 2010; Fenouil et al. 2012; Liang et al. 2017). This was based on our analysis that revealed correlations between gene expression, nucleosome landscape, and chromatin accessibility of CGI promoters (Supplemental Figs. S1A, S3A). To test this hypothesis, we first considered whether RNA polymerase II-dependent gene transcription contributes to the nucleosome landscape or accessibility of gene promoters in a manner opposing that of PRC1. We used the chemical inhibitor triptolide to acutely inhibit RNA polymerase II initiation and occupancy prior to performing ATAC-seq (Fig. 5A–D; Supplemental Fig. S7A). This resulted in significant increases in nucleosome occupancy (Fig. 5B,C) and decreased distances between nucleosomal dyads in the triptolide-treated cells compared to their untreated control (Fig. 5D), with the nucleosome landscape of PcG-free promoters in triptolide-treated ESCs now more closely resembling PcG-bound sites in untreated cells. This was consistent with RNA polymerase II countering the activity of PRC1 at gene promoters. However, it also suggested that the changes we observed at gene promoters in the PRC1-deficient cells might be linked to their transcriptional reactivation and not simply removal of the PRC1 complex. To examine this possibility, we performed nuclear RNA-seq in the *Ring1*^−/−^;*Rnf2*^*fl/fl*^ and *Ring1*^−/−^;*Rnf2*^*fl/f*^;*Eed*^−/−^ ESCs before and after tamoxifen treatment to identify gene promoters that were activated following removal of PRC1 and/or PRC2 and directly compared these effects to alterations in the nucleosome landscape. Differential gene expression analysis identified 11.2%, 14.1%, and 21.2% of all CGI promoters, and 35.6%, 36.1%, and 59.2% of PcG target genes, with significant increases in gene expression after loss of PRC1, PRC2, or PRC1/2, respectively, with a high degree of overlap between cell lines and treatments (Fig. 5E,F). We then compared the accessibility and nucleosome landscape at the promoters of activated genes with those whose expression was unaffected by the loss of PcG complexes. Consistent with our previous analysis, there were very few significant changes in ATAC-seq signal, and these did not correlate with altered transcriptional activity at gene promoters in any of the PRC-null cell lines or triptolide-treated cells (Fig. 5G; Supplemental Fig. S7B–E), demonstrating that promoter chromatin accessibility is not dependent on the transcriptional state and must be established by other mechanisms. When we examined the nucleosome occupancy and spacing at PcG-bound promoters activated in the absence of PRC1, there were larger decreases in nucleosome occupancy and increased distances between nucleosome dyads compared to PcG-occupied promoters whose expression levels remained unchanged (Fig. 5H–K). Although this was consistent with transcriptional changes potentially shaping the nucleosome landscape instead of a direct contribution from PRC1, we then examined PcG-bound promoters with increased activity in the PRC2-null cells. We reasoned that if RNA polymerase II and not PRC1 was responsible for the changes in the nucleosome landscape, one would expect to see comparable changes in the nucleosome landscape at up-regulated gene promoters in the PRC2-null cells. However, this was not the case, because PcG target genes activated in the PRC2-null cells showed negligible or very minor differences in their nucleosome occupancy or spacing at their promoters compared to promoters with unaltered activity (Fig. 5I–K). This therefore demonstrates that although RNA polymerase II activity can influence the nucleosome landscape, it is not sufficient to explain the changes that we observed at reactivated genes in the PRC1-null cells. This suggests that even in the presence of elevated transcriptional activity in PRC2-null cells, PRC1 may restrain the nucleosome landscape at these sites, potentially through disrupting RNA polymerase II-dependent chromatin remodeling. This represents a new distinction between how PRC1 and PRC2 function to shape chromatin organization at PcG chromatin domains.

## Discussion

It has been proposed that PcG complexes establish and maintain a transcriptionally repressive chromatin state at gene promoters. In vitro biochemical experiments demonstrated that PcG complexes can compact chromatin (Francis et al. 2004; Trojer et al. 2007, 2011; Grau et al. 2011), and PcG-occupied gene promoters display reduced accessibility in vivo compared to non-PcG promoters (Fig. 1; Zink and Paro 1995; McCall and Bender 1996; Boivin and Dura 1998; Fitzgerald and Bender 2001; Bell et al. 2010; Calabrese et al. 2012; Beck et al. 2014; Deaton et al. 2016). Here, we discover that PRC1, but not PRC2, is required to maintain a chromatin landscape that is characterized by elevated nucleosome occupancy and more closely spaced nucleosomes (Figs. 2–4). Unexpectedly, the ability of PRC1 to influence the local nucleosome landscape was not required to maintain the less accessible chromatin state characteristic of PcG-occupied promoters, demonstrating that chromatin compaction of reconstituted nucleosomes in vitro by PcG complexes cannot explain the limited accessibility at PcG target sites in vivo. Therefore, measurement of the accessibility and other features of the chromatin landscape at PcG targets appear not to be directly coupled, consistent with previous reports in other experimental systems (Mieczkowski et al. 2016; Mueller et al. 2017). Furthermore, we reveal that the nucleosome landscape associated with loss of PRC1 is linked to reactivation of the associated gene and may potentially reflect altered RNA polymerase II activity in the absence of PRC1 (Fig. 5). Importantly, the relationship between transcriptional activation and altered nucleosome landscape was not observed in PRC2-null cells, highlighting that the transcriptionally repressive function of PRC1, but not PRC2, is linked to increased nucleosome occupancy and closer packing of nucleosomes. Together our new observations have broad implications for understanding PcG-dependent gene repression and the relationship between chromatin accessibility, the nucleosome landscape and transcriptional activity at gene promoters.

Some of the earliest studies examining chromatin at PcG-occupied sites reported reduced accessibility compared to regulatory sites lacking PcG proteins. It has been proposed that PcG protein occupancy on chromatin may define this less accessible state (McCall and Bender 1996; Fitzgerald and Bender 2001; Bell et al. 2010; Calabrese et al. 2012; Beck et al. 2014; Deaton et al. 2016). However, this possibility has not been systematically examined at the genome scale in vivo. Here, we directly test whether PcG proteins define accessibility at PcG-occupied gene promoters and unexpectedly find no causal relationship between the occupancy of PcG proteins and chromatin accessibility, a conclusion that was also recently reported in an independent study (Hodges et al. 2018). Therefore other activities must define the lack of accessibility at PcG genes. One possible explanation for this reduced accessibility could be that PcG target genes in ESCs have an underrepresentation of tissue-specific transcription factor binding sites, which are often implicated in the recruitment of chromatin remodeling complexes such as BAF (SWI/SNF) (Ku et al. 2008; Mendenhall et al. 2010; Hu et al. 2011; Guertin and Lis 2013; Marathe et al. 2013; King and Klose 2017). In agreement with this possibility, it was recently shown that synthetic recruitment of BAF to a PcG-occupied gene promoter resulted in eviction of PcG proteins and increased accessibility (Kadoch et al. 2017). Because we and others have shown that loss of PcG proteins is not sufficient to cause increases in chromatin accessibility at PcG target genes (Hodges et al. 2018), this increase in accessibility was presumably dependent on the chromatin remodeling activity of the BAF complex and suggests that limited activity of BAF or possibly other chromatin remodeling complexes may explain the low accessibility of PcG-occupied chromatin. Another feature of PcG-occupied promoters is their low levels of histone acetylation compared to other gene promoters. Histone acetylation is associated with elevated chromatin accessibility (Rincon-Arano et al. 2012; Lennartsson et al. 2015; Frank et al. 2016). At PcG targets, the removal of acetylation from lysine residues in histone tails would reinstate their positive charge and allow them to more stably interact with DNA and possibly limit accessibility (Norton et al. 1989; Shogren-Knaak et al. 2006). This could be mediated by the nucleosome remodeling and deacetylase (NuRD) complex that co-occupies many PcG target sites (Yildirim et al. 2011; Reynolds et al. 2012) and has been demonstrated to limit chromatin accessibility at regulatory elements (Ramírez et al. 2012; de Dieuleveult et al. 2016). Ultimately, it remains unclear what defines reduced chromatin accessibility at PcG targets, and what, if any, role this plays in regulating gene expression at these sites.

Our analysis of the nucleosome landscape at PcG target sites revealed a role for PRC1 in regulating nucleosome occupancy and spacing that is distinct from chromatin accessibility. One would have predicted that the reduced occupancy of nucleosomes following removal of PRC1 would yield an increase in chromatin accessibility, but this is not evident in our analysis. The precise molecular explanation for this discord remains unclear. One possibility is that alternative proteins engage with sites vacated by PcG proteins competing with nucleosomes for occupancy, but not affecting overall accessibility measurements. Alternatively, the effects we observe on the nucleosome landscape in PRC1-null cells are very subtle and may not lead to a profound enough perturbation of local chromatin structure to manifest in overall increases in chromatin accessibility. Nevertheless, a lack of concordance between the measurement of accessibility and nucleosome features has been reported previously (Mieczkowski et al. 2016; Mueller et al. 2017), indicating that the relationship between these measurements is not always simple to rationalize. Clearly in future work it will be important to understand in more detail how the nucleosome landscape of gene promoters is related to measurements of chromatin accessibility, particularly in the context of PcG-bound sites.

Here, we have disrupted PRC1 by removing the core scaffolding proteins RING1/RNF2 which are also the E3 ubiquitin ligases required for deposition of H2AK119ub1. PRC1 has been proposed to function through E3 ligase-dependent and ligase-independent activities (Endoh et al. 2012; Blackledge et al. 2014; Cooper et al. 2014; Illingworth et al. 2015; Pengelly et al. 2015; Rose et al. 2016) and its ability to compact chromatin in vitro is thought to be independent of its ubiquitin ligase activity (Francis et al. 2004; Margueron et al. 2008). It will be interesting to determine if these E3 ligase-independent activities characterized in vitro contribute to PRC1's effect on the nucleosome landscape in vivo by examining PcG-occupied chromatin in situations in which the E3 ligase activity of RING1/RNF2 has been eliminated (Endoh et al. 2012; Illingworth et al. 2015). However, if the catalytic activity of PRC1 is not responsible for shaping the nucleosome landscape, how could this be achieved? Two PRC1 components linked to chromatin compaction and the inhibition of chromatin remodeling in vitro, BMI1 (formerly known as PCGF4) and CBX2, contain highly basic and disordered protein domains that are conserved across different PcG components in different species (Grau et al. 2011; Beh et al. 2012). Increasing the acidity of this domain in CBX2 disrupted its ability to inhibit chromatin remodeling (Grau et al. 2011), suggesting that the presence of these basic and highly charged domains might also be important for the in vivo regulation of nucleosome occupancy and spacing. However, both CBX2 and BMI1 are expressed at low levels in ESCs and form only a small minority of PRC1 complexes (Kloet et al. 2016), so it is unclear what their contribution toward the PcG-dependent nucleosome landscape could be in this cell type. Finally, several studies support the possibility that PRC1 might interfere directly with RNA polymerase II occupancy or activity (Stock et al. 2007; Brookes et al. 2012; Lehmann et al. 2012). In agreement with these findings, following deletion of PRC1, we observed that a subset of promoters is susceptible to transcriptional activation and these then acquire a nucleosome landscape consistent with elevated RNA polymerase II activity. Alterations in the nucleosome landscape following PRC1 removal are therefore likely driven by processes linked to transcription. However, elevated transcription per se is not necessarily sufficient to drive these outcomes, because some PcG target genes display elevated expression following removal of PRC2, yet nevertheless retain a PRC1-dependent nucleosome landscape. Investigating the detailed mechanisms that define the nucleosome landscape at PcG target genes and how this is related to gene transcription will be an interesting area for future work and will be fundamental to understanding how PcG complexes repress gene transcription.

In conclusion, we have discovered that PRC1 can influence the nucleosome landscape at PcG target genes in a manner that does not contribute to reduced chromatin accessibility. This indicates that PRC1-dependent chromatin compaction observed in vitro does not explain the reduced accessibility at PcG target sites in vivo and reveals a new and previously unappreciated complexity in the relationship between PcG complexes, the nucleosome landscape, and gene repression.

## Methods

### Cell culture and lines

Mouse embryonic stem cell (ESC) lines were grown on gelatin-coated plates in DMEM supplemented with 15% FBS, 10 ng/mL leukemia-inhibitory factor, penicillin/streptomycin, β-mercaptoethanol, L-glutamine, and nonessential amino acids. *Ring1*^−/−^;*Rnf2*^*fl/fl*^ ESCs (Endoh et al. 2008) were adapted to grow under feeder-free culture conditions and were treated with 800 nM 4-hydroxytamoxifen (TAM) for 72 h to ablate RNF2 levels. EED conditional knockout ESCs that express a doxycycline-sensitive *Eed4* transgene (*Eed4*^TG^) in an *Eed*^−/−^ background were treated with 1 µg/mL doxycycline (DOX) for 14 d to disrupt PRC2 complex and function, as previously described (Ura et al. 2008; Tavares et al. 2012). SV40-immortalized *Ring1*^−/−^;*Rnf2*^*fl/fl*^ mouse embryonic fibroblasts (MEFs) (Endoh et al. 2012; Jullien et al. 2017) were grown in DMEM supplemented with 7% FBS and penicillin/streptomycin and maintained in culture for up to 10 passages. *Ring1*^−/−^;*Rnf2*^*fl/fl*^ MEFs were treated with 800 nM TAM for 96 h to ablate RNF2 levels. Loss of protein expression and Polycomb complex activity was verified by Western blotting using the following antibodies: RNF2 (Blackledge et al. 2014), SMARCA4 (Abcam, ab110641), EED (Millipore, #09-774), HDAC1 (Abcam, ab109411), H2AK119ub1 (Cell Signalling Technology [CST] #8240), H3K27me3 (Diagenode, pAb-069-050) (Rose et al. 2016), and H3 (Farcas et al. 2012), H2A (CST, #3636), and H4 (CST, #2935). All cell lines were confirmed to be mycoplasma-free.

### Generation of Polycomb double-knockout ESCs

To delete EED in the *Ring1*^−/−^;*Rnf2*^*fl/fl*^ ESCs, CRISPR/Cas9 guides were designed flanking exons 2 to 5 of *Eed* (Guide 1: 5′-CACCGACAATCAGTGCTCTTACTCG-3′; Guide 2: 5′-CACCGAAACAGTAAGAGTCGAGTCG-3′) to induce a frameshift in all four EED translation products. The *Eed* sgRNAs were cloned into pSpCas9(BB)-2A-Puro (plasmid 48139; Addgene) using a previously described protocol (Ran et al. 2013). Lipofectamine 3000 (Life Technologies) was used to transfect Cas9-sgRNA plasmids into *Ring1*^−/−^;*Rnf2*^*fl/fl*^ ESCs, and transfected cells were treated with 1 µg/mL puromycin for 48 h. After 10 d, individual colonies were isolated, expanded, and genomic DNA was screened by PCR for deletion of *Eed* exons 2 to 5 (FWD: 5′-AGCAGGCAGATACCAGAGTG-3′; REV 5′-ATGTCAGCACGTCCCAACTA-3′). Putative *Eed*^−/−^ clones were confirmed by Western blotting. *Ring1*^−/−^;*Rnf2*^*fl/fl*^;*Eed*^−/−^ cells were treated with 800 nM TAM for 72 h to ablate RNF2 expression.

### Inhibition of RNA polymerase II

To inhibit RNA polymerase II activity, E14 ESCs were preplated at 2.5 × 10^6^ cells/10 cm plate and allowed to grow for 24 h prior to treatment with 500 nM triptolide (TRP) for 50 min, as previously described (Jonkers et al. 2014). To limit reactivation of RNA polymerase II, cells were immediately washed with ice-cold PBS and harvested by cell scraping prior to nuclei isolation for RNA and ATAC analysis. To validate TRP treatment, real-time reverse transcriptase PCR was performed using intronic (pre-mRNA) primer sequences and normalized to the RNA polymerase III-transcribed U6 snRNA gene using the ΔΔCt method. Global run-on sequencing (GRO-seq) data from an identical triptolide treatment of mouse ESCs was obtained from GSE48895 (Jonkers et al. 2014).

### ATAC-seq sample preparation and sequencing

Chromatin accessibility was assayed using an adaptation of the assay for transposase accessible chromatin (ATAC)-seq (Buenrostro et al. 2013), as previously described (King and Klose 2017). Briefly, nuclei were isolated in 1 mL HS Lysis buffer (50 mM KCl, 10 mM MgSO_4_.7H_2_O, 5 mM HEPES, 0.05% NP-40 [IGEPAL CA630]), 1 mM PMSF, 3 mM DTT) for 1 min at room temperature and washed three times with ice-cold RSB buffer (10 mM NaCl, 10 mM Tris [pH 7.4], 3 mM MgCl_2_). Next, 5 × 10^4^ nuclei were counted and resuspended in 1× Tn5 reaction buffer (10 mM TAPS, 5 mM MgCl_2_, 10% dimethylformamide) with 2 µL Tn5 transposase (25 µM) made in house according to the previously described protocol (Picelli et al. 2014). Reactions were incubated for 30 min at 37°C before isolation and purification of tagmented DNA using QIAquick MinElute columns (Qiagen). ATAC-seq libraries were prepared by PCR amplification using single index (i7) Illumina barcodes previously described (Buenrostro et al. 2013) and the NEBNext High-Fidelity 2× PCR Master Mix with 8–10 cycles. Libraries were quantified by qPCR using SensiMix SYBR (Bioline) and KAPA Library Quantification DNA standards (KAPA Biosystems) and sequenced on Illumina NextSeq 500 using 80-bp paired-end reads in biological duplicate (*Eed*^−/−^;*Eed4.TG*^*DOX*^), triplicate (TRP treatment and *Ring1*^−/−^;*Rnf2*^*fl/fl*^ MEFs), or quadruplicate (*Ring1*^−/−^;*Rnf2*^*fl/fl*^ and *Ring1*^−/−^;*Rnf2*^*fl/fl*^;*Eed*^−/−^).

### MNase-seq sample preparation and sequencing

For micrococcal nuclease (MNase)-seq experiments, we used an adaptation of a native ChIP protocol described previously (Rose et al. 2016). Briefly, nuclei were isolated from 5 × 10^7^ *Ring1*^−/−^;*Rnf2*^*fl/fl*^ mouse embryonic stem cells with or without TAM treatment with RSB buffer (10 mM Tris-HCl [pH 8], 10 mM NaCl, 3 mM MgCl_2_) supplemented with 0.1% NP-40 and 5 mM N-ethylmaleimide. This was followed by digestion for 5 min at 37°C with 16 units MNase (Fermentas) in 1 mL RSB supplemented with 0.25 M sucrose, 3 mM CaCl_2_, and 10 mM N-ethylmaleimide. After digestions were stopped with 4 mM EDTA, nuclei were pelleted by centrifugation at 1500*g* and the soluble S1 fraction collected. Pelleted nuclei were then resuspended in 300 µL nucleosome release buffer (10 mM Tris-HCl [pH 7.5], 10 mM NaCl, 0.2 mM EDTA, 10 mM N-ethylmaleimide), incubated for 1 h at 4°C with gentle rotation, and then gently passed through a 27G syringe needle five times. After the insoluble material was pelleted by centrifugation at 1500*g*, the soluble S2 fraction was collected and combined with the S1 fraction. To prepare material for constructing sequencing libraries, DNA was purified from chromatin corresponding to 5 × 10^6^ cells using ChIP DNA Clean and Concentrator kit (Zymo). The efficiency of MNase digestion was assessed by DNA electrophoresis (1.5% agarose gel). MNase-seq libraries were prepared from 500 ng DNA using the NEBNext Ultra II DNA Library Prep Kit (NEB) according to the manufacturer's protocol. DNA fragment size selection step was included to enrich for mononucleosome size fragments in the final libraries. Libraries were quantified as for ATAC-seq libraries and were sequenced on Illumina NextSeq 500 using 80-bp paired-end reads in biological triplicate.

### Nuclear RNA-seq sample preparation and sequencing

To purify nuclear RNA, nuclei were isolated as described for ATAC-seq prior to resuspension in TRIzol reagent (Thermo Fisher Scientific) and RNA extraction according to the manufacturer's protocol. RNA was treated with the TURBO DNA-free Kit (Thermo Fisher Scientific), and rRNA was depleted using the NEBNext rRNA Depletion kit (NEB). RNA-seq libraries were prepared using the NEBNext Ultra Directional RNA-seq kit (NEB), and libraries were sequenced on the Illumina NextSeq 500 with 80-bp paired-end reads in biological quadruplicate.

### Sequencing data alignment, processing and normalization

For mouse ESC ATAC-seq, MNase-seq, DNase-seq (GSE37074) (Yue et al. 2014), FAIRE-seq (GSE49141) (Thakurela et al. 2013), Tn5 digestion control (GSE87822) (King and Klose 2017), RNF2 and SUZ12 ChIP-seq (GSE83135) (Rose et al. 2016), and Bio-CAP-seq (GSE43512) (Long et al. 2013), paired-end reads were aligned to the mouse mm10 genome using Bowtie 2 (Langmead and Salzberg 2012) with the “--no-mixed” and “--no-discordant” options, and single-end libraries were aligned using default Bowtie 2 settings. Nonuniquely mapping reads and reads mapping to a custom blacklist of artificially high regions of the genome were discarded. For RNA-seq, reads were initially aligned using Bowtie 2 against the rRNA genomic sequence (GenBank: BK000964.3) to quantify and filter out rRNA fragments prior to alignment against the mm10 genome using the STAR RNA-seq aligner (Dobin et al. 2012). PCR duplicates were removed using SAMtools (Li et al. 2009). Biological replicates were randomly down-sampled to contain the same number of reads for each individual replicate and merged to create a representative genome track using DANPOS2 (Chen et al. 2013) for ATAC-seq and MNase-seq samples, MACS2 (Zhang et al. 2008) for ChIP-seq, FAIRE-seq, and Bio-CAP-seq, or genomeCoverageBed (Quinlan 2014) for RNA-seq. Genome coverage tracks were visualized using the UCSC Genome Browser (Kent et al. 2002).

### Differential accessibility and gene expression analysis

Significant changes in ATAC-seq data sets were identified using the DiffBind package (http://bioconductor.org/packages/release/bioc/vignettes/DiffBind/inst/doc/DiffBind.pdf); for RNA-seq, DESeq2 (Love et al. 2014) was used with a custom-built, nonredundant mm10 gene set (Rose et al. 2016). For DiffBind analysis of ATAC-seq data sets, *FDR* < 0.05 and a fold change >1.5-fold was deemed a significant change, whereas for DESeq2 analysis of RNA-seq a threshold of *FDR* < 0.05 and a fold change greater than twofold was used.

### Annotation and analysis of Polycomb target sites

Nonredundant RefGene TSS intervals (±500 bp; *n* = 20,633) were overlapped with mouse ESC RNF2 and SUZ12 peak sets previously identified from biological triplicate data with input control using MACS2 (Rose et al. 2016), and any TSS overlapping with both RNF2 and SUZ12 were considered to be bona fide Polycomb target TSSs. Nonmethylated CpG island (CGI) intervals were experimentally identified in ESCs using MACS2 peak calling of Bio-CAP-seq (Blackledge et al. 2012; Long et al. 2013), and only TSSs within CGI intervals were used for subsequent promoter-based analyses. For MEF and mouse ENCODE tissues, CGI intervals downloaded from the UCSC Genome Browser that overlapped with the nonredundant set of TSSs were annotated with whole-genome bisulfite sequencing (GSE42836) (Hon et al. 2013) or reduced representation bisulfite sequencing (GSE52741) (Hu et al. 2014) derived methylation calls lifted over to mm10 using the liftOver tool from UCSC (Hinrichs et al. 2006), and only intervals with methylation <5% were considered. For each tissue or cell line, Polycomb target CGI TSSs were identified by overlapping with ENCODE H3K27me3 peaks (GSE49847) (Yue et al. 2014) lifted over from mm9 to mm10 or identified from MEF H3K27me3 ChIP-seq (GSE91374) (Han et al. 2017) using MACS2 peak calling. To identify Polycomb-bound distal regulatory elements in mouse ESCs, ATAC peaks identified with DANPOS2 in wild-type ESCs were annotated with H3K4me1 (GSE27844) (Whyte et al. 2012) and H3K4me3 (GSE49847) (Yue et al. 2014) to classify putative distal regulatory elements as previously described (King and Klose 2017), and peaks overlapping with both RNF2 and SUZ12 were considered Polycomb targets. Gene expression-matched promoters were identified using untreated *Ring1*^−/−^;*Rnf2*^*fl/fl*^ ESC nuclear RNA-seq normalized expression values calculated by DESeq2. Metaplot analysis of ATAC-seq, MNase-seq, ChIP-seq, or nucleosome occupancy profiles at gene promoters was performed using HOMER (Heinz et al. 2010). Quantitation of reads per kilobase per million (RPKM) was performed within CGI intervals at TSSs using custom scripts (Supplemental Code). Data were visualized using R (v 3.4.1) (R Core Team 2017) and ggplot2 (Wickham 2009), with scatterplots colored by density using stat_density2d. Regression and correlation analyses were also performed in R using standard linear models and Pearson correlation, respectively.

### Characterization of nucleosome features at gene promoters

As a simple measure of nucleosome occupancy at promoters, the fragment sizes of Tn5-tagmented DNA fragments within each promoter interval were extracted from ATAC-seq .bam files and used to calculate the median fragment size per CGI promoter interval (Supplemental Code). Higher median fragment sizes correspond to higher levels of nucleosome-sized Tn5-tagmented DNA, whereas lower fragment sizes correspond to higher levels of nucleosome-free DNA. To complement this approach, we extracted signal corresponding to nucleosome occupancy and positional information within CGI promoters using the NucleoATAC package (Schep et al. 2015), which relies upon a model-based analysis of Tn5 tagmentation fragment size profiles to reflect the probability of nucleosome occupancy at a given loci. Importantly, both methods are independent of the total coverage of tagmented fragments (i.e., accessibility) at different loci. For MNase-seq data sets, nucleosome positions and occupancy were determined using DANPOS2 (Chen et al. 2013). In order to visualize nucleosome occupancy, we profiled the occ.bedgraph files from our NucleoATAC analysis and normalized .wig for MNase-seq tracks centered upon TSSs in 1-bp resolution and identified average nucleosome positions using the local maxima of the coverage. Quantification of total nucleosome occupancy per kb for CGI promoters was performed by calculating the coverage of NucleoATAC-derived .occ.bedgraph files using BEDtools “coverage” tool (Quinlan 2014) or the median nucleosome summit height from DANPOS2 MNase-seq nucleosome calls per CGI (Supplemental Code). Individual nucleosome dyad centers identified in the nucmap_combined.bed file from NucleoATAC or MNase-seq nucleosome calls from DANPOS2 were used to calculate the distance to the nearest neighboring nucleosome dyad center (inter-dyad distance) using the BEDtools “closest” tool (Supplemental Code). Only nucleosomes within CGI intervals were included for this analysis, and the median inter-dyad distances for each CGI interval were calculated. Median nucleosome fuzziness scores per CGI were calculated from NucleoATAC-derived nucpos.bed files or DANPOS2 MNase-seq nucleosome calls (Supplemental Code).

## Data access

The ATAC-seq, MNase-seq, and RNA-seq data from this study have been submitted to the NCBI Gene Expression Omnibus (GEO; https://www.ncbi.nlm.nih.gov/geo/) under accession number GSE98403. Custom scripts used for analysis are provided as Supplemental Code and are available at https://github.com/hamishking/gff-annotation-tools.

## Supplementary Material

Supplemental Material
