# Supplementary material for: Polycomb repressive complex 1 shapes the nucleosome landscape but not accessibility at target genes
Source: Genome Res. 2018 Oct;28(10):1494–507. doi: 10.1101/gr.237180.118 (PMC6169895; doi:10.1101/gr.237180.118)
Supplement: Supplemental Material [file supp_28_10_1494__index.html]

Polycomb repressive complex 1 shapes the nucleosome landscape but not accessibility at target genes — Supplemental Material 

# Polycomb repressive complex 1 shapes the nucleosome landscape but not accessibility at target genes

## Supplemental Material

- Supplemental\_Figures.pdf
- Supplemental\_Code.zip
