# Supplementary material for: Polycomb repressive complex 1 shapes the nucleosome landscape but not accessibility at target genes
Source: Genome Res. 2018 Oct;28(10):1494–507. doi: 10.1101/gr.237180.118 (PMC6169895; doi:10.1101/gr.237180.118)
Supplement: Supplemental Material [file supp_gr.237180.118_Supplemental_Figures.pdf]

Supplemental Figure S1

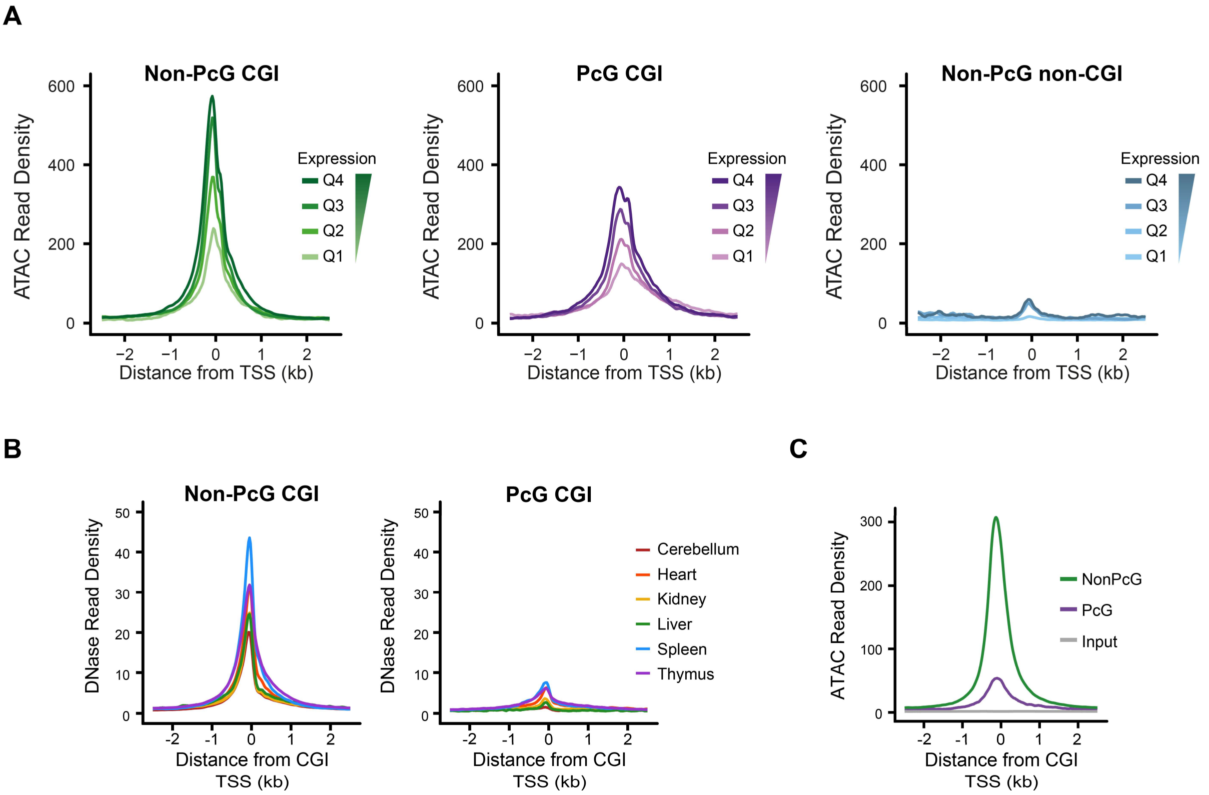

Supplemental Figure S1. Analysis of chromatin accessibility at Polycomb and non-Polycomb CGI promoters.

A) A metaplot analysis of wild type mouse ESC ATAC-seq profiles at non-PcG CGI, PcG-bound CGI and non-CGI PcG-free promoters at different gene expression quartiles (Q1 lowest -> Q4 highest), centred on the TSS.

B) A metaplot analysis of ENCODE DNase-seq for different mouse tissues at H3K27me3-positive or H3K27me3-negative non-methylated CGI promoters, centred on the TSS.

C) A metaplot analysis of wild type mouse embryonic fibroblast ATAC-seq profiles at H3K27me3-positive (PcG; *n* = 1438) or H3K27me3-negative CGI promoters (Non-PcG; *n* = 10118), centred on the TSS.

Supplemental Figure S2

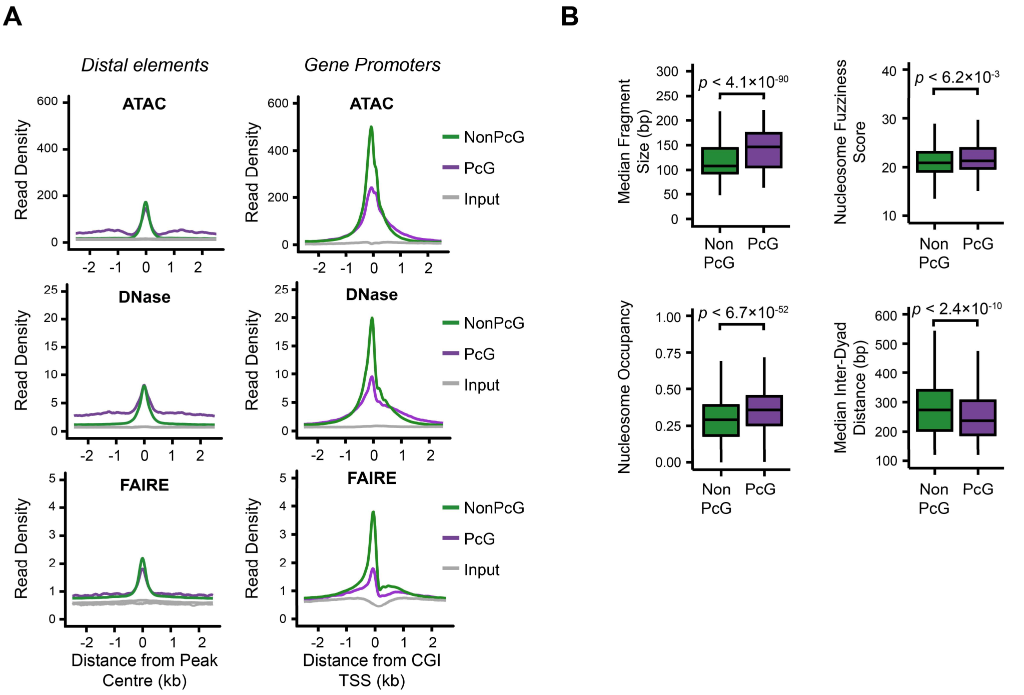

Supplemental Figure S2. Chromatin accessibility and nucleosome landscape features at distal elements bound by Polycomb.

A) A metaplot analysis of ATAC-seq, DNase-seq and FAIRE-seq signal at PcG-occupied (*n* = 1229) or PcG-free (*n* = 43134) distal elements (left) compared with CGI promoters (right; as in 1D).

B) Boxplots comparing the median ATAC-seq fragment sizes, NucleoATAC-derived nucleosome occupancy signal, nucleosome fuzziness and median inter-dyad distances for PcG-occupied (n = 4020) or non-PcG (n = 10251) CGI promoters.

Supplemental Figure S3

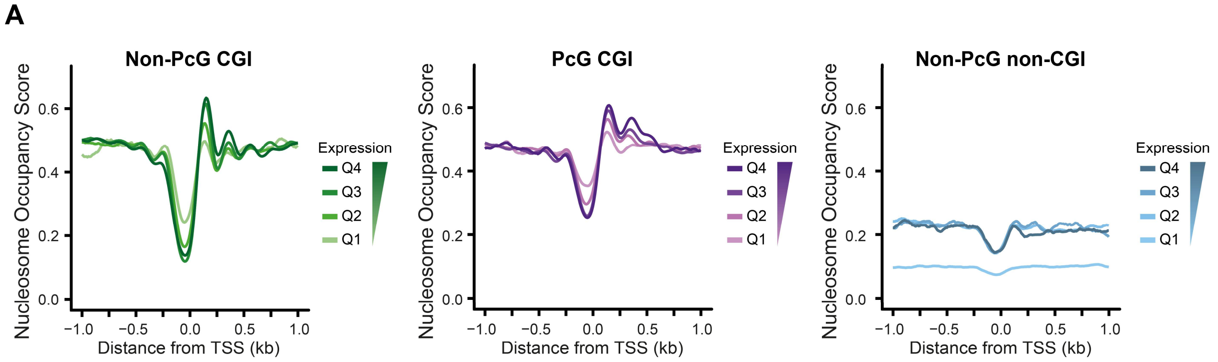

Supplemental Figure S3. Nucleosome landscape of expression-matched PcG-bound or PcG-free promoters.

A) A metaplot analysis of wild type mouse ESC NucleoATAC-derived nucleosome occupancy scores at non-PcG CGI, PcG-bound CGI and non-CGI PcG-free promoters of different gene expression quartiles (Q1 lowest -> Q4 highest).

Supplemental Figure S4

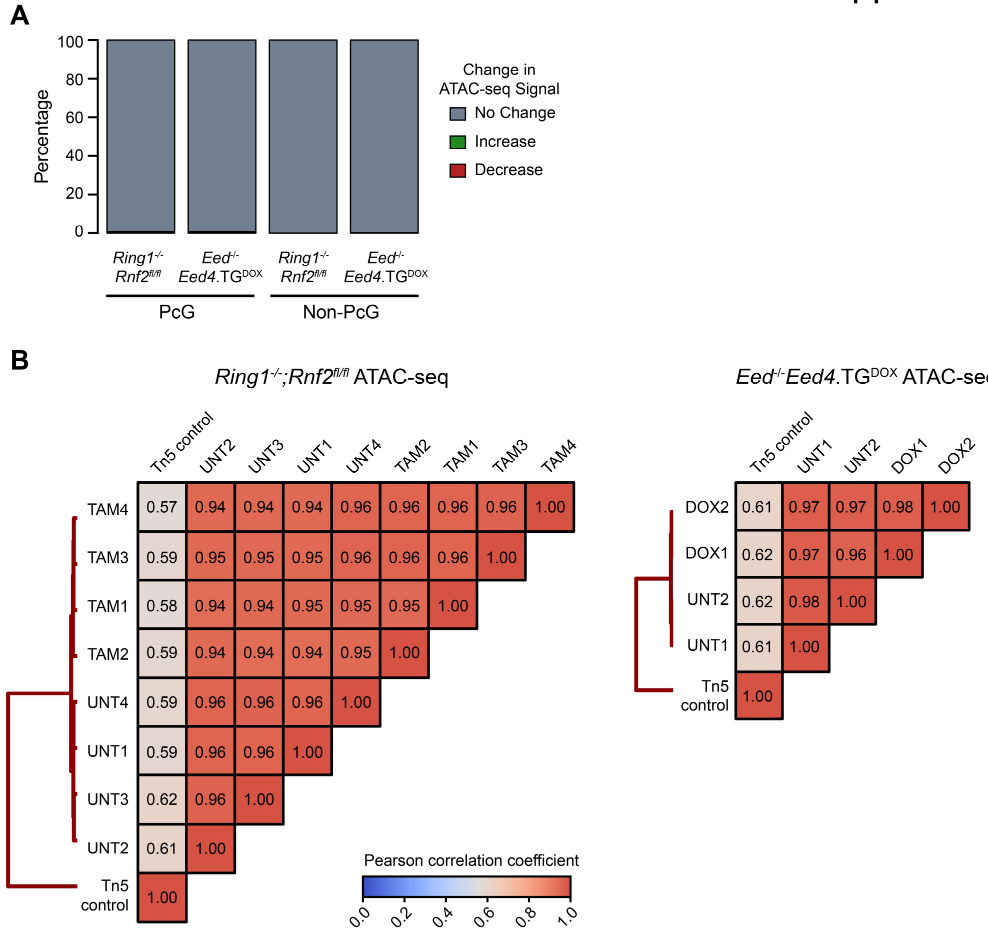

Supplemental Figure S4. Statistical analysis and reproducibility of ATAC-seq datasets.

A) A barplot depicting the number of significant changes in ATAC-seq signal at PcG or Non-PcG CGI promoters after ablation of PRC1 (*Ring1*$^{-/-}$;*Rnf2*$^{fl/fl}$) or PRC2 (*Eed*$^{-/-}$;*Eed4.TG*^{DOX}).

B) Pearson correlation matrices for biological replicate ATAC-seq signal at wild type ESC ATAC hypersensitive sites for *Ring1*$^{-/-}$;*Rnf2*$^{fl/fl}$ and *Eed*$^{-/-}$;*Eed4.TG*^{DOX} ATAC-seq experiments.

Supplemental Figure S5

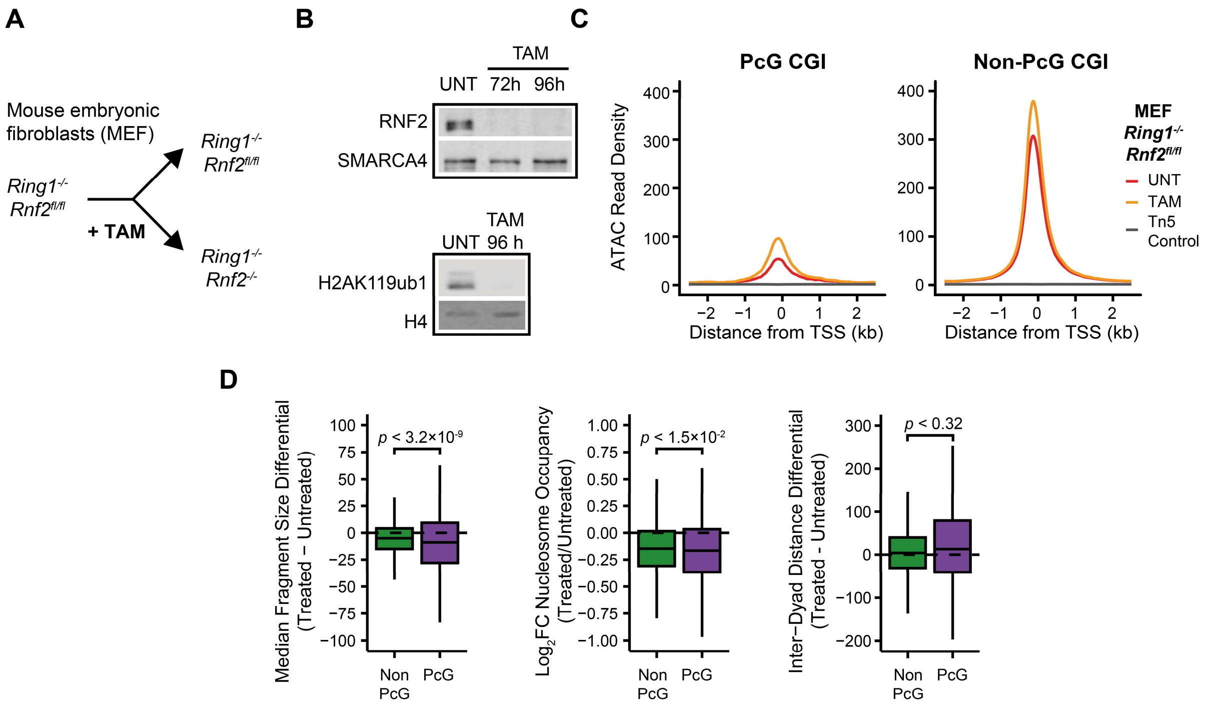

Supplemental Figure S5. Analysis of chromatin accessibility and the nucleosome landscape in PRC1-null mouse embryonic fibroblasts.

A) A schematic depicting the treatment of *Ring1*$^{-/-}$;*Rnf2*$^{fl/fl}$ mouse embryonic fibroblasts (MEFs) with 4-hydroxytamoxifen (TAM) to generate PRC1-null MEFs.

B) A Western blot analysis of untreated and TAM-treated *Ring1*$^{-/-}$;*Rnf2*$^{fl/fl}$ MEFs for RNF2 (upper) and H2AK119ub1 (lower) at different time points. 96 hours after TAM treatment was used for all future experiments.

C) A metaplot analysis for *Ring1*$^{-/-}$;*Rnf2*$^{fl/fl}$ MEF ATAC-seq before and after tamoxifen (TAM) treatment at H3K27me3-positive (PcG; *n* = 1438) or H3K27me3-negative CGI promoters (Non-PcG; *n* = 10118).

D) Quantitation of differences in median ATAC-seq fragment sizes, NucleoATAC-derived nucleosome occupancy, and inter-dyad distances for PcG and non-PcG CGI promoters in *Ring1*$^{-/-}$;*Rnf2*$^{fl/fl}$ MEF ATAC-seq before and after TAM treatment.

Supplemental Figure S6

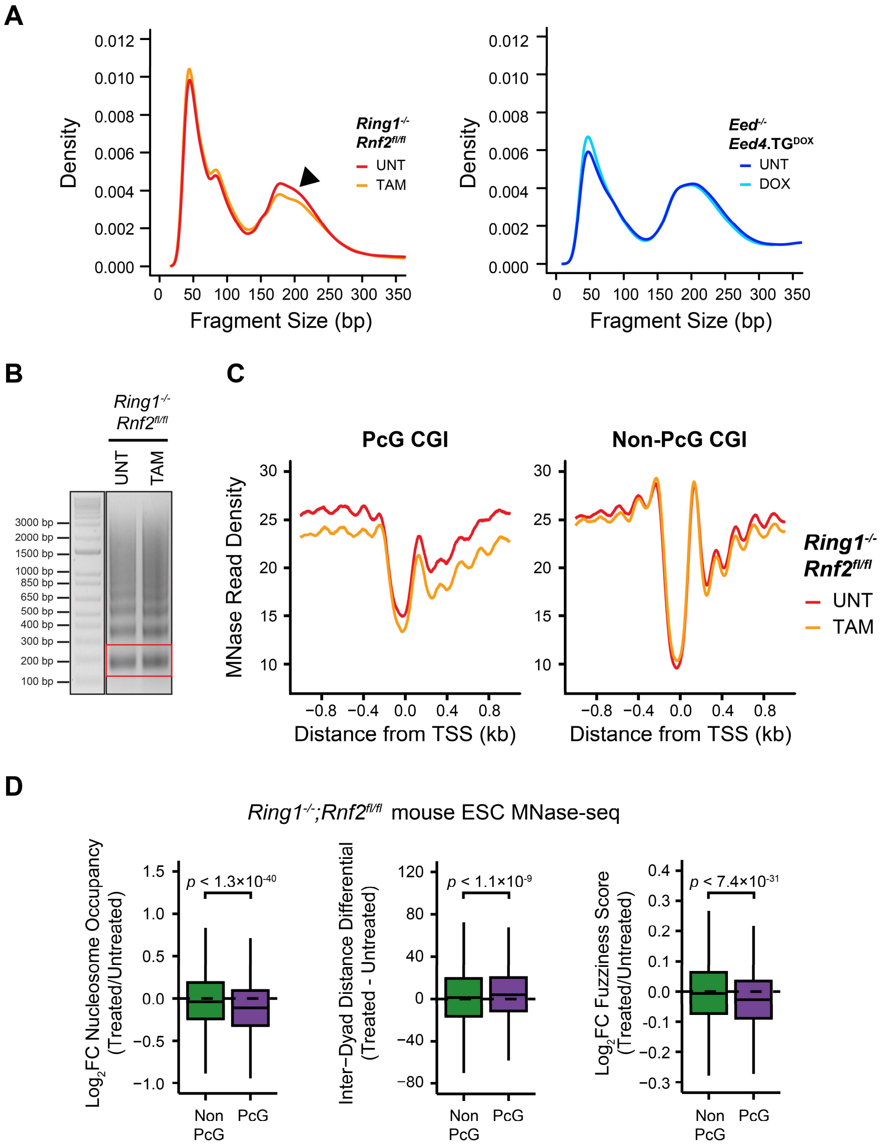

Supplemental Figure S6. Deletion of PRC1 results in an altered nucleosome landscape.

A) Frequency distribution plots for ATAC-seq fragment sizes in PcG-occupied CGI promoter intervals in *Ring1*$^{-/-}$;*Rnf2*$^{fl/fl}$ and *Eed*$^{-/-}$;*Eed4.TG*^{DOX} ESC ATAC-seq with or without tamoxifen (TAM) or doxycycline (DOX) treatment respectively. The arrow highlights loss of mono-nucleosome-sized fragments in the *Ring1*$^{-/-}$;*Rnf2*$^{fl/fl}$ experiment.

B) An exemplar agarose gel (1.5 %) electrophoresis of MNase-digested native chromatin of a single replicate of MNase-seq library construction for *Ring1*$^{-/-}$;*Rnf2*$^{fl/fl}$ ESCs. The red box highlights the mono-nucleosome fraction that was size-selected during library preparation.

C) A metaplot analysis for *Ring1*$^{-/-}$;*Rnf2*$^{fl/fl}$ MNase-seq before and after TAM treatment at PcG-occupied (n = 4020) or PcG-free (n = 10251) CGI promoters centred on the TSS.

D) Comparison of differences in MNase-seq derived measurements of nucleosome occupancy, inter-dyad distance and nucleosome fuzziness for PcG and non-PcG CGI promoters in *Ring1*$^{-/-}$;*Rnf2*$^{fl/fl}$ ESCs before and after TAM treatment.

Supplemental Figure S7

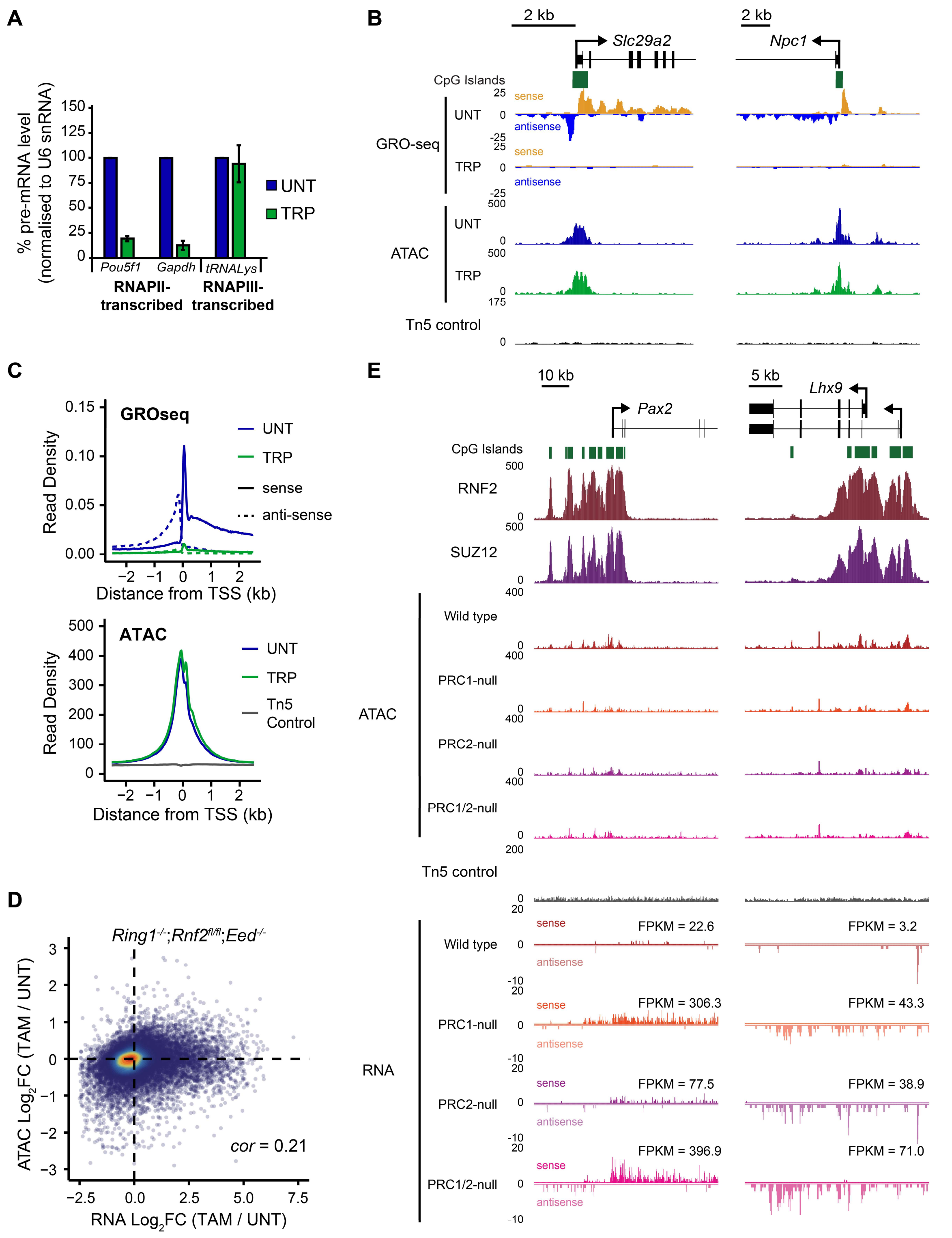

Supplemental Figure S7. Promoter accessibility is independent of transcriptional activity.

A) Quantitative real-time reverse transcriptase PCR for *Pou5f1*, *Gapdh* and *tRNA-Lys* genes following treatment of mouse E14 ESCs with 500 nM triptolide (TRP) for 50 min, normalised to expression of U6 snRNA and untreated control cells. *n* = 3 ± stdev.

B) A genome screenshot at two gene promoters depicting RNA polymerase II engagement (GRO-seq; Jonkers et al. 2014) and ATAC-seq following TRP treatment.

C) A metaplot analysis for GRO-seq and ATAC-seq data following TRP treatment at CGI TSS (n = 14271).

D) A scatterplot comparing the log2 fold change in gene expression (RNA) and chromatin accessibility (ATAC) for CGI promoters in the *Ring1*$^{-/-}$;*Rnf2*$^{fl/fl}$;*Eed*$^{-/-}$ ESCs after tamoxifen (TAM) treatment.

E) A genome screenshot for *Ring1*$^{-/-}$;*Rnf2*$^{fl/fl}$ and *Ring1*$^{-/-}$;*Rnf2*$^{fl/fl}$;*Eed*$^{-/-}$ ATAC-seq and nuclear RNA-seq signal before and after TAM at two PcG target genes, *Pax2* and *Lhx9*, that are up-regulated after loss of PRC1 and/or PRC2. The normalised expression values (FPKM) for each gene in each cell line and treatment are annotated.
